# Prediction of Japan Score and Society of Thoracic Surgeons Risk Scores for Patients Undergoing Cardiovascular Surgery by Serum Growth Differentiation Factor-15 and Endothelin-1 Levels

**DOI:** 10.7759/cureus.82508

**Published:** 2025-04-18

**Authors:** Taira Fukuda, Takashi Kato, Yuta Kanazawa, Rina Hirai, Hayato Ishizaka, Hideaki Tan, Ikuko Shibasaki, Hirotsugu Fukuda, Shigeru Toyoda, Toshiaki Nakajima

**Affiliations:** 1 Department of Health and Nutrition, Faculty of Human Sciences, University of Human Arts and Sciences, Saitama, JPN; 2 Department of Cardiovascular Surgery, School of Medicine, Dokkyo Medical University, Mibu, JPN; 3 Department of Rehabilitation, Dokkyo Medical University Hospital, Mibu, JPN; 4 Department of Cardiovascular Medicine, School of Medicine, Dokkyo Medical University, Mibu, JPN

**Keywords:** adiponectin, endothelin-1, gdf-15, japan score, sts score

## Abstract

Background: The Society of Thoracic Surgeons surgical risk score (STS Score) is widely used to assess risk in cardiac surgery along with the Japan Score (Japan surgical risk score) in conjunction with the various biomarkers.

Methods: Preoperative blood tests were performed on 127 patients who subsequently underwent cardiovascular surgery (78 men, 49 women; mean age, 69.5 years). Serum levels of growth differentiation factor (GDF)-15, endothelin-1, and adiponectin were measured by enzyme-linked immunosorbent assay and used as independent variables in multivariate regression analysis with the STS Score or Japan Score as the dependent variable.

Results: Serum levels of GDF-15, endothelin-1, and adiponectin correlated positively with the STS Score, the Japan Score, and the plasma brain natriuretic peptide (BNP) level, and correlated negatively with the estimated glomerular filtration rate (eGFR) and hemoglobin and albumin levels. After adjustment for age, sex, and body mass index, multivariate linear regression analysis showed that log (GDF-15) defined log (STS Score), and log (GDF-15) and log (endothelin-1) were independent factors defining log (Japan Score) (GDF-15: β = 0.372, p < 0.001; endothelin-1: β = 0.213, p = 0.016). eGFR and levels of hemoglobin and albumin defined log (GDF-15), and log (BNP) defined log (endothelin-1).

Conclusions: The STS Score and the Japan Score were associated with serum GDF-15 level in patients undergoing cardiovascular surgery. It is likely that the Japan Score had a stronger association with heart failure than the STS Score.

## Introduction

Cardiovascular surgery remains high risk, despite recent breakthroughs in surgical techniques [1,2], and preoperative risk assessment with surgical risk scores is important. The Society of Thoracic Surgeons (STS) surgical risk score (STS Score) (https://alexioncalculator.research.sts.org/riskcalc/) is widely used to assess risk in cardiac surgery and has high accuracy in predicting short- and long-term mortality risk [3,4]. The Japanese Cardiovascular Surgery Database (JCVSD) was established in 2000 with the cooperation of the STS, and online data registration began in Japanese in 2001 [5]. The Japan Score, which is the Japan surgical risk score based on the Japan Congenital Cardiovascular Surgery Database (JCVSD) (https://jcvsd.org/JapanSCORE/), can predict mortality, 30-day mortality, and risk of major complications [6,7].

Blood biomarkers measured preoperatively are useful in predicting postoperative risk [8-10]. Endothelin-1, a 21-amino acid endothelium-derived vasoconstrictor peptide [11], has attracted attention as a potential therapeutic target for the treatment of a number of cardiovascular diseases with the use of endothelin-1 receptor antagonists [12,13]. Preoperative plasma endothelin-1 levels are predictive of postoperative acute kidney injury (AKI) [8], and elevated plasma endothelin-1 levels are strongly associated with pulmonary hypertension, heart failure, and mortality [14]. Growth differentiation factor (GDF)-15, a stress-responsive member of the transforming growth factor-β cytokine superfamily [15,16], is an independent determinant of prognosis in healthy subjects [17,18] and patients with heart failure [19]. Our previous study showed that preoperative levels of GDF-15 and endothelin-1, as well as intraoperative factors, such as cardiopulmonary bypass time, may help identify the short-term surgical risk for patients undergoing cardiovascular surgery [9]. Furthermore, elevated blood adiponectin level (normal reference value is 0.87-21.42 μg/mL) was a negative prognostic determinant in patients with advanced heart failure [20,21]. While both surgical risk scores and serum biomarkers are used in preoperative risk assessment, the specific relationship between them is not fully understood. For example, it is not clear which serum biomarkers are most predictive of specific surgical risk scores or how changes in biomarker levels correlate with changes in risk score categories. This gap in knowledge limits the ability to personalize risk assessment and optimize preoperative management [22].

This study aimed to clarify associations between the STS Score and the Japan Score and serum levels of GDF-15 and endothelin-1 and adiponectin in patients who subsequently underwent cardiovascular surgery. We hypothesized that the Japan Score was more strongly associated with a heart failure-related biomarker, endothelin-1, compared with the STS Score, since the Japan Score includes a heart failure item (presence of acute heart failure within two weeks).

## Materials and methods

Participants

Patients undergoing cardiovascular surgery (N = 127; 78 men, 49 women; mean age, 69.5 years) were enrolled. The inclusion criteria were as follows: (1) patients undergoing elective cardiovascular procedures, defined as those scheduled in advance and excluding emergency or urgent cases; (2) procedures including coronary artery bypass grafting (CABG), valve replacement or repair, combined CABG and valve procedures, and aortic surgery; and (3) adult patients aged ≥18 years with a confirmed diagnosis of cardiovascular disease requiring surgical intervention. The exclusion criteria were as follows: (1) patients with cerebrovascular disease or those undergoing arthroscopic surgery; (2) patients with severe orthopedic diseases, malignancies, or chronic conditions including cognitive impairment; (3) patients undergoing emergency surgery; and (4) patients with incomplete preoperative data or severe comorbidities contraindicating surgery. This study was conducted in adherence to the ethical guidelines of the Declaration of Helsinki and was approved by the Ethics Committee of Dokkyo Medical University Hospital, which was the place of this study (approval date: October 13, 2015; approval number: 27074). Informed consent was obtained from all participants prior to the start of the study.

Data collection

The data collected included age, sex, measured height and weight, comorbidities, and left ventricular ejection fraction (LVEF). Clinical chemistry and hematological parameters, including hemoglobin (Hb), albumin (Alb), high-sensitivity C-reactive protein (hsCRP), hemoglobin A1c (HbA1c), brain natriuretic peptide (BNP), and estimated glomerular filtration rate (eGFR), were assessed using standard chemical methods in the clinical laboratory of Dokkyo Medical University Hospital. To measure fasting serum GDF-15, endothelin-1, and adiponectin levels, peripheral venous blood was drawn into pyrogen-free tubes without ethylenediaminetetraacetic acid on the morning of the cardiovascular surgery. The serum was stored in aliquots at -80°C for enzyme-linked immunosorbent assay (ELISA).

STS Score

The STS Score includes the following items: basic patient information (age, sex, weight and height, body mass index (BMI)), smoking history, medical history (diseases affecting surgical risk), diabetes mellitus (with or without insulin therapy), hypertension, stroke (presence or absence of prior stroke), pulmonary disease (presence or absence of chronic obstructive pulmonary disease (COPD)), renal disease (presence or absence of dialysis, degree of renal dysfunction), and myocardial infarction (recent history, especially whether within six hours). It also includes heart condition: LVEF, severity of heart failure (according to the New York Heart Association (NYHA) classification), angina (at rest or on exertion), myocardial ischemia (with or without recent episode), valvular disease (degree of stenosis or regurgitation), and presence or absence of pulmonary hypertension; and surgical situation: surgical urgency (emergency or planned surgery), type of surgery (CABG, valve replacement, valve repair, complex surgery, etc.), presence or absence of reoperation (history of previous cardiac surgery), and presence or absence of aortic dissection or aortic aneurysm. Furthermore, it includes other risk factors, such as infection (e.g., active endocarditis), liver disease (including severity), and peripheral arterial disease.

Japan Score

The Japan Score includes the following items: basic patient information: age, sex, weight and height (BMI), medical history and comorbidities, diabetes mellitus (with or without insulin therapy), chronic renal failure (with or without dialysis), pulmonary disease (COPD), presence or absence of atrial fibrillation, and cerebrovascular disease (history of stroke or a transient ischemia attack (TIA)). A TIA, often called a “mini-stroke,” is a temporary disruption of blood flow to the brain, causing stroke-like symptoms that usually resolve within an hour, without causing permanent brain damage. It also includes cardiac function (LVEF, heart failure (NYHA classification), presence or absence of acute heart failure within two weeks, and presence or absence of pulmonary hypertension) and surgical situation (surgical urgency (emergency surgery or not) and preoperative infection (e.g., endocarditis)).

ELISA

Serum GDF-15 levels were measured by GDF-15 Quantikine ELISA Kit (DGD150, R&D Systems, Inc., Minneapolis, MN, USA) as previously described [9,10]. The mean intra-assay coefficient of variation (CV) was 2.3%, and the inter-assay CV was 5.4%. The detection threshold of GDF-15 was 0.004 ng/mL. Serum endothelin-1 levels were measured by the endothelin-1 Quantikine ELISA Kit (DET100, R&D Systems, Inc., Minneapolis, MN, USA) as previously described [9]. The mean intra-assay CV was 2.7%, and the inter-assay CV was 6.3%. The detection threshold of endothelin-1 was 0.207 pg/mL. Serum adiponectin levels were measured by adiponectin Quantikine ELISA Kit (DRP300, R&D Systems, Inc., Minneapolis, MN, USA). The mean intra-assay CV was 3.7%, and the inter-assay CV was 6.8%. The detection threshold was 0.0009 μg/mL. Samples, reagents, and buffers were prepared according to the manufacturer’s recommendations.

Statistical analysis

Data are presented as mean ± standard deviation (SD), median (interquartile range (IQR)), and number (%). After testing for normality (Kolmogorov-Smirnov test), we evaluated the associations between parameters with Pearson’s correlation coefficients in the case of normally distributed parameters or Spearman’s correlation coefficients in the case of non-normally distributed parameters. Multivariate linear regression analyses were performed as dependent variables of surgical risk scores (STS Score and Japan Score) and as independent variables of serum biomarkers (GDF-15, endothelin-1, and adiponectin). Also, multivariate linear regression analyses were performed as dependent variables of serum biomarkers (GDF-15 and endothelin-1) and as independent variables of clinical data (Hb, Alb, hsCRP, HbA1c, BNP, eGFR, and LVEF). Age, sex, and BMI were used as covariates for all analyses. When the actual measured values for both dependent and independent variables did not follow a normal distribution, the data were logarithmically transformed to achieve a normal distribution. The absence of multicollinearity among variables was defined as a variance inflation factor (VIF) of <3. All statistical analyses were performed using IBM SPSS Statistics for Windows, Version 28 (Released 2021; IBM Corp., Armonk, New York, United States). p < 0.05 was regarded as significant.

## Results

The median (IQR) age of the participants was 72.0 (66.0-78.0) years, and the median BMI was 23.2 (21.3-26.0) kg/m2. The types of surgery performed included CABG (n = 29), valve surgery (n = 58), aortic surgery (n = 9), and combined procedures (n = 31). The median serum GDF-15 level was 1.28 (0.80-2.33) ng/mL, and the median serum endothelin-1 level was 1.10 (0.93-1.53) pg/mL. The median serum adiponectin level was 7.42 (3.59-13.60) μg/mL. The median STS Score was 2.48 (1.17-4.50), and the median Japan Score was 2.90 (1.60-6.10). Values for clinical laboratory tests are summarized in Table 1.

**Table 1:** Participant parameters SD, standard deviation; IQR, interquartile range; BMI, body mass index; eGFR, estimated glomerular filtration rate; Hb, hemoglobin; hsCRP, high-sensitivity C-reactive protein; HbA1c, hemoglobin A1c; Alb, albumin; BNP, brain natriuretic peptide; LVEF, left ventricular ejection fraction; GDF, growth differentiation factor; STS, the Society of Thoracic Surgeons; CKD, chronic kidney disease; CABG, coronary artery bypass graft Total (n = 127). Data are shown as mean ± SD, median (IQR), and number (%)

Variable	Mean ± SD, median (IQR), or number (%)
Age, years	72.0 (66.0-78.0)
Male	78 (61)
Female	49 (39)
BMI, kg/m^2^	23.2 (21.3-26.0)
eGFR, mL/min/1.73 m^2^	57.9 ± 27.0
Hb, g/dL	12.4 ± 1.9
hsCRP, mg/L	0.15 (0.04-0.55)
HbA1c, %	5.9 (5.5-6.4)
Alb, g/dL	4.0 (3.6-4.3)
BNP, pg/mL	173 (65-467)
LVEF, %	61.3 (51.3-66.3)
GDF-15, ng/mL	1.28 (0.80-2.33)
Endothelin-1, pg/mL	1.10 (0.93-1.53)
Adiponectin, μg/mL	7.42 (3.59-13.60)
STS Score	2.48 (1.17-4.50)
Japan Score	2.90 (1.60-6.10)
Hypertension	94 (74)
Diabetes mellitus	42 (33)
Dyslipidemia	59 (46)
Smoking	18 (14)
CKD	65 (51)
Hemodialysis	13 (10)
CABG	29 (23)
Valve surgery	58 (43)
Aortic surgery	9 (7)
Combined procedure	31 (24)

**Figure 1:**
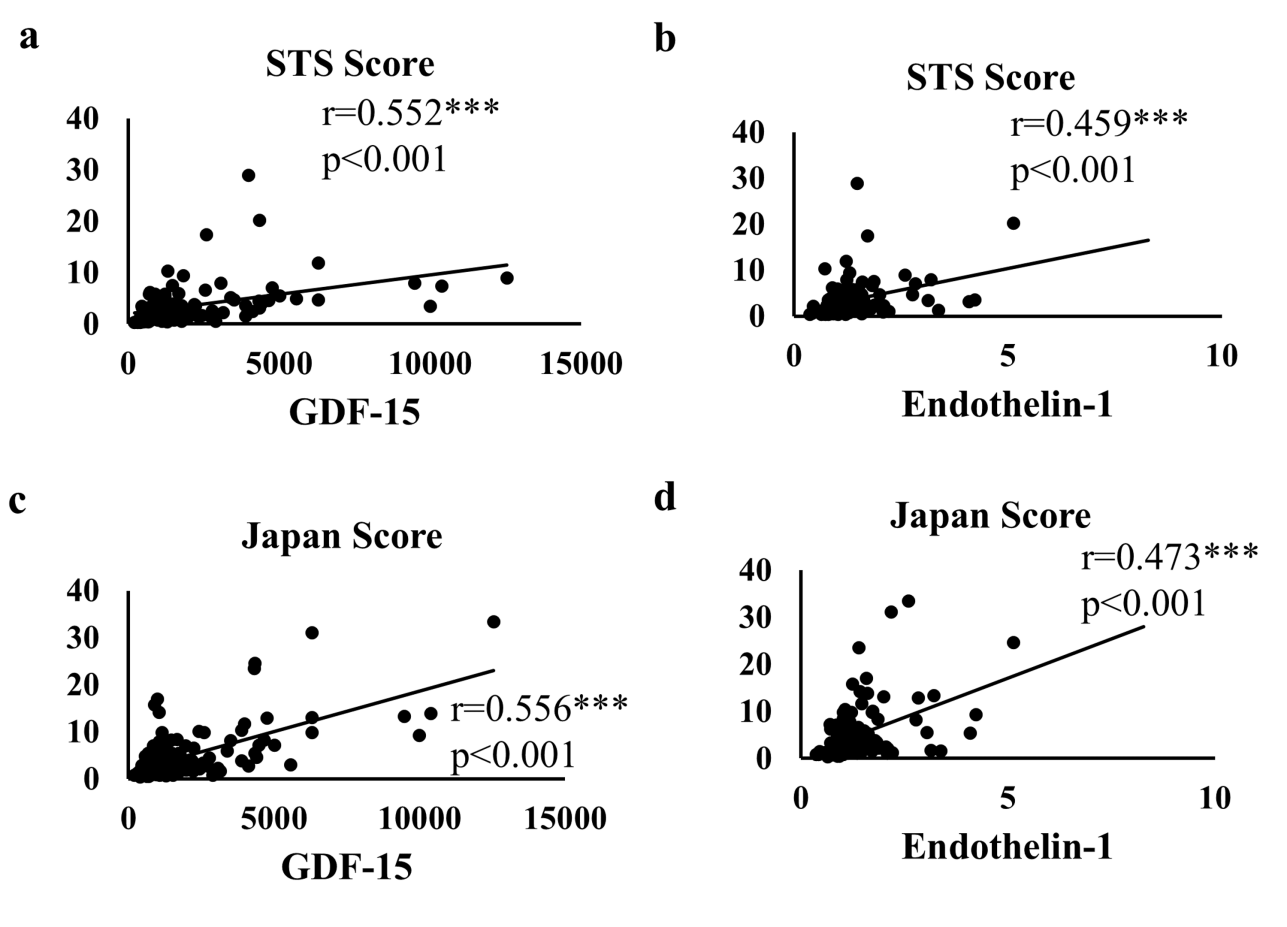
Correlation between the surgical risk scores and levels of preoperative biomarkers in patients undergoing cardiovascular surgery GDF, growth differentiation factor; STS, the Society of Thoracic Surgeons Correlations between the STS Score and serum GDF-15 levels (a), STS Score and serum endothelin-1 levels (b), Japan Score and serum GDF-15 levels (c), and Japan Score and serum endothelin-1 levels (d)

Serum levels of GDF-15 (r = 0.552, p < 0.001; Figure 1a), endothelin-1 (r = 0.459, p < 0.001; Figure 1b), and adiponectin (r = 0.612, p < 0.001) correlated with the STS Score. Serum levels of GDF-15 (r = 0.556, p < 0.001; Figure 1c), endothelin-1 (r = 0.473, p < 0.001; Figure 1d), and adiponectin (r = 0.398, p < 0.001) also correlated with the Japan Score.

**Figure 2:**
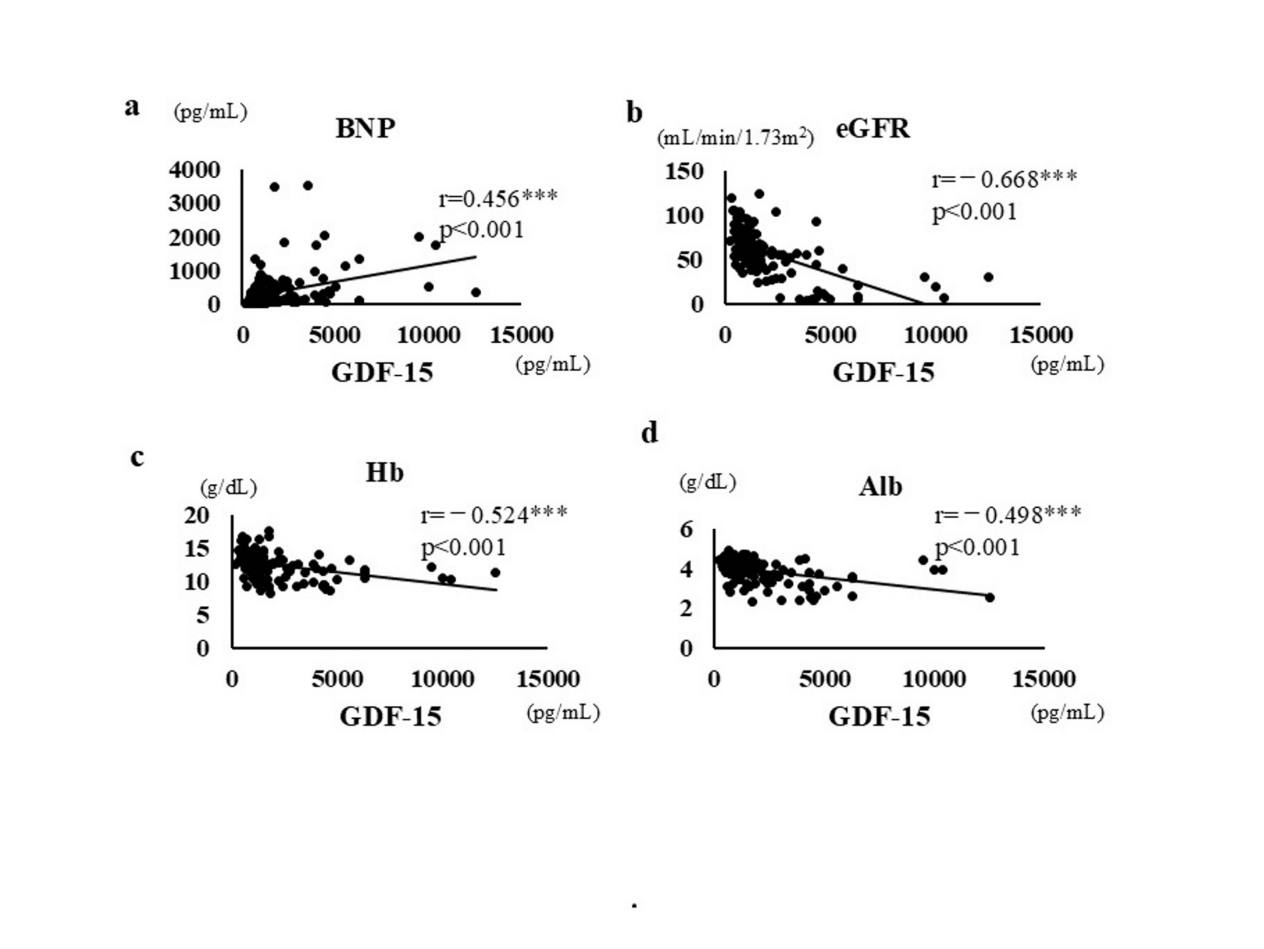
Correlation between clinical data and preoperative serum GDF-15 levels in patients undergoing cardiovascular surgery GDF, growth differentiation factor; BNP, brain natriuretic peptide; eGFR, estimated glomerular filtration rate; Hb, hemoglobin; Alb, albumin Correlations between the plasma BNP level (a), eGFR (b), and levels of Hb (c) and Alb (d) and serum GDF-15 levels

Serum GDF-15 levels correlated positively with plasma BNP levels (r = 0.456, p < 0.001, Table 2, Figure 2a) and negatively with eGFR (r = -0.668, p < 0.001, Figure 2b), Hb levels (r = -0.524, p < 0.001, Figure 2c), and serum Alb levels (r = -0.498, p < 0.001, Figure 2d). Serum endothelin-1 levels correlated positively with plasma BNP levels (r =0.660, p < 0.001) and negatively with eGFR (r = -0.419, p < 0.001), Hb levels (r = -0.668, p < 0.001), and serum Alb levels (r = -0.335, p < 0.001). Serum adiponectin levels correlated positively with plasma BNP levels (r = 0.629, p < 0.001) and negatively with eGFR (r = -0.292, p = 0.001), Hb levels (r = -0.392, p < 0.001), and serum Alb levels (r = -0.271, p = 0.003). The correlation matrix of relationships of values of clinical laboratory tests with serum levels of GDF-15, endothelin-1, and adiponectin is presented in Table 2.

**Table 2:** Correlation matrix of relationships of participant parameters with serum levels of GDF-15, endothelin-1 and adiponectin GDF, growth differentiation factor; BMI, body mass index; eGFR, estimated glomerular filtration rate; Hb, hemoglobin; hsCRP, high-sensitivity C-reactive protein; HbA1c, hemoglobin A1c; Alb, albumin; BNP, brain natriuretic peptide; LVEF, left ventricular ejection fraction; STS, Society of Thoracic Surgeons Data are shown as r-value (p-value). * p < 0.05, ** p < 0.01, *** p < 0.001

Variable	GDF-15	Endothelin-1	Adiponectin
	r-value (p-value)	r-value (p-value)	r-value (p-value)
Age	0.352 (<0.001)***	0.140 (0.118)	0.429 (<0.001)***
Sex	-0.154 (0.085)	-0.102 (0.255)	0.243 (0.008)**
BMI	-0.216 (0.015)*	-0.148 (0.099)	-0.330 (<0.001)***
eGFR	-0.668 (<0.001)***	-0.419 (<0.001)***	-0.292 (0.001)**
Hb	-0.524 (<0.001)***	-0.277 (0.002)**	-0.392 (<0.001)***
hsCRP	0.250 (0.005)**	0.100 (0.268)	0.078 (0.398)
HbA1c	0.092 (0.310)	0.110 (0.227)	-0.192 (0.039) *
Alb	-0.498 (<0.001)***	-0.335 (<0.001)***	-0.271 (0.003) **
BNP	0.456 (<0.001)***	0.660 (<0.001)***	0.629 (<0.001)***
LVEF	-0.200 (0.032)*	-0.252 (0.006)**	-0.155 (0.108)
STS Score	0.552 (<0.001)***	0.459 (<0.001)***	0.612 (<0.001)***
Japan Score	0.556 (<0.001)***	0.473 (<0.001)***	0.398 (<0.001)***
GDF-15	Not applicable	0.545 (<0.001)***	0.409 (<0.001)***
Endothelin-1	0.545 (<0.001)***	Not applicable	0.392 (<0.001)***
Adiponectin	0.409 (<0.001)***	0.392 (<0.001)***	Not applicable

Multivariate linear regression analysis showed that log (GDF-15) and log (adiponectin) were independent factors determining log (STS Score) (GDF-15: β = 0.361, p < 0.001, adiponectin: β = 0.385, p < 0.001, Table 3). It also found that log (GDF-15) remained an independent factor determining log (STS Score) after adjusting for age, sex, and BMI (β = 0.375, p < 0.001).

**Table 3:** Multivariate linear regression analysis of relationship between STS Score and serum levels of GDF-15, endothelin-1 and adiponectin GDF, growth differentiation factor; STS, Society of Thoracic Surgeons; VIF, variance inflation factor Model 1, univariate analysis adjusted by age, sex and BMI; Model 2, multivariate analysis, unadjusted; Model 3, multivariate analysis adjusted by age, sex and BMI.　Data are shown as β-value (p-value). * p < 0.05, ** p < 0.01, *** p < 0.001

Dependent variable: log STS Score	Model 1	Model 2	Model 3	VIF
Adjusted R^2^	Not applicable	0.442	0.624	
Independent variable	β-value (p-value)	β-value (p-value)	β-value (p-value)	Model 2/Model 3
GDF-15 (log)	0.536 (<0.001)***	0.361 (<0.001)***	0.375 (<0.001)***	1.590/1.890
Endothelin-1 (log)	0.407 (<0.001)***	0.067 (0.464)	0.149 (0.054)	1.570/1.614
Adiponectin (log)	0.346 (<0.001)***	0.385 (<0.001)***	0.128 (0.109)	1.336/1.726

In addition, it showed that log (GDF-15) was an independent factor determining log (Japan Score) (β = 0.420, p < 0.001, Table 4) and that log (GDF-15) and log (endothelin-1) were independent factors determining log (Japan Score) after adjusting for age, sex, and BMI (GDF-15: β = 0.372, p < 0.001; endothelin-1: β = 0.213, p = 0.016).

**Table 4:** Multivariate linear regression analysis of relationship between Japan Score and serum levels of GDF-15, endothelin-1, and adiponectin GDF, growth differentiation factor; VIF, variance inflation factor Model 1, univariate analysis adjusted by age, sex, and BMI; Model 2, multivariate analysis, unadjusted; Model 3, multivariate analysis adjusted by age, sex, and BMI. Data are shown as β-value (p-value). * p < 0.05, ** p < 0.01, *** p < 0.001

Dependent variable: log Japan Score	Model 1	Model 2	Model 3	VIF
Adjusted R^2^	Not applicable	0.372	0.426	
Independent variable	β-value (p-value)	β-value (p-value)	β-value (p-value)	Model 2/Model 3
GDF-15 (log)	0.570 (<0.001)***	0.420 (<0.001)***	0.372 (<0.001)***	1.563/1.889
Endothelin-1 (log)	0.446 (<0.001)***	0.165 (0.070)	0.213 (0.016)*	1.504/1.546
Adiponectin (log)	0.292 (0.002)**	0.163 (0.054)	0.058 (0.519)	1.291/1.600

Further, multivariate linear regression analysis showed that eGFR, Hb level, and serum Alb level were independent factors determining log (GDF-15) (eGFR: β = -0.540, p < 0.001; Hb: β = -0.194, p = 0.010; Alb: β = -0.290, p = 0.003, Table 5). It also found that eGFR, Hb level, and serum Alb level remained independent factors determining log (GDF-15) after adjusting for age, sex, and BMI (eGFR: β = -0.545, p < 0.001; Hb: β = -0.210, p = 0.007; Alb: β = -0.192, p = 0.043).

**Table 5:** Multivariate linear regression analysis of relationship between serum levels of GDF-15 and clinical data GDF, growth differentiation factor; BMI, body mass index; eGFR, estimated glomerular filtration rate; Hb, hemoglobin; hsCRP, high-sensitivity C-reactive protein; HbA1c, hemoglobin A1c; Alb, albumin; BNP, brain natriuretic peptide; LVEF, left ventricular ejection fraction; VIF, variance inflation factor Model 1, univariate analysis adjusted by age, sex, and BMI; Model 2, multivariate analysis, unadjusted; Model 3, multivariate analysis adjusted by age, sex, and BMI. Data are shown as β-value (p-value). * p < 0.05, ** p < 0.01, *** p < 0.001

Dependent variable: log GDF-15	Model 1	Model 2	Model 3	VIF
Adjusted R^2^	Not applicable	0.611	0.654	
Independent variable	β-value (p-value)	β-value (p-value)	β-value (p-value)	Model 2 / Model 3
eGFR	-0.630 (<0.001)***	-0.540 (<0.001)***	-0.545 (<0.001)***	1.208 / 1.293
Hb	-0.436 (<0.001)***	-0.194 (0.010)**	-0.210 (0.007)**	1.564 / 1.898
hsCRP (log)	0.288 (<0.001)***	-0.026 (0.733)	0.032 (0.668)	1.636 / 1.825
HbA1c	0.093 (0.260)	0.046 (0.447)	0.040 (0.480)	1.038 / 1.057
Alb	-0.438 (<0.001)***	-0.290 (0.003)**	-0.192 (0.043)*	2.646 / 2.852
BNP (log)	0.415 (<0.001)***	0.061 (0.439)	0.005 (0.953)	1.770 / 2.034
LVEF	-0.116 (0.188)	0.032 (0.635)	0.045 (0.492)	1.323 / 1.405

It also showed that log (BNP) was an independent factor defining log (endothelin-1) (β = 0.522, p < 0.001, Table 6) and that log (BNP) remained an independent factor defining log (endothelin-1) after adjusting for age, sex, and BMI (β = 0.574, p < 0.001).

**Table 6:** Multivariate linear regression analysis of relationship between serum levels of endothelin-1 and clinical data GDF, growth differentiation factor; BMI, body mass index; eGFR, estimated glomerular filtration rate; Hb, hemoglobin; hsCRP, high-sensitivity C-reactive protein; HbA1c, hemoglobin A1c; Alb, albumin; BNP, brain natriuretic peptide; LVEF, left ventricular ejection fraction; VIF, variance inflation factor Model 1, univariate analysis adjusted by age, sex, and BMI; Model 2, multivariate analysis, unadjusted; Model 3, multivariate analysis adjusted by age, sex, and BMI. Data are shown as β-value (p-value). * p < 0.05, ** p < 0.01, *** p < 0.001

Dependent variable: log Endothelin-1	Model 1	Model 2	Model 3	VIF
Adjusted R^2^	Not applicable	0.296	0.312	
Independent variable	β-value (p-value)	β-value (p-value)	β-value (p-value)	Model 2/Model 3
eGFR	-0.319 (<0.001)***	-0.081 (0.351)	-0.095 (0.289)	1.208/1.293
Hb	-0.221 (0.021)*	0.041 (0.679)	-0.061 (0.573)	1.564/1.898
hsCRP (log)	0.088 (0.325)	-0.097 (0.337)	-0.138 (0.192)	1.636/1.825
HbA1c	0.091 (0.317)	0.111 (0.171)	0.102 (0.205)	1.038/1.057
Alb	-0.273 (0.003)**	-0.062 (0.631)	-0.029 (0.827)	2.646/2.852
BNP (log)	0.633 (<0.001)***	0.522 (<0.001)***	0.574 (<0.001)***	1.770/2.034
LVEF	-0.254 (0.007)**	-0.025 (0.781)	0.026 (0.782)	1.323 / 1.405

## Discussion

The results of this study showed that even after adjusting for age, sex, and BMI, the GDF-15 level defined the STS Score, and GDF-15 and endothelin-1 levels were independent factors defining the Japan Score in patients undergoing cardiovascular surgery. In addition, eGFR, Hb, and Alb defined the GDF-15 level, and BNP defined the endothelin-1 level. Thus, the Japan Score was suggested to have a stronger association with heart failure than the STS Score.

JCVSD was established in 2000 with the cooperation of STS, and online data registration began in Japanese in 2001 [5]. In 2008, the Japan Score was introduced and used to predict mortality risk in adult cardiovascular surgery [6]. The Japan Score can calculate predicted mortality, 30-day mortality, and risk of major complications [6,7]. In thoracic aortic surgery, the 30-day mortality rate predicted by the Japan Score was 6.7%, and the 30-day operative mortality rate predicted by the Japan Score was 8.6%, while the incidence of stroke and renal failure requiring dialysis was 6.1% and 6.7%, respectively [23]. These results were consistent with surgical estimates of high risk (>5%) in aortic and major vascular surgery [1]. The risk of mortality was found to be particularly high among patients undergoing emergency surgery. The area under the receiver operating characteristic curve (AUC) was found to be as high as 0.79 and 0.78 for 30-day and surgical mortality, respectively [23]. Furthermore, for CABG alone, the 30-day and operative mortality rates predicted by the Japan Score were 2.0% and 2.7%, respectively [6]. Risk factors such as emergency surgery, preoperative creatinine level >3.0 mg/dL, aortic valve stenosis, and obstructive lung disease showed high odds ratios, and AUC was 0.85, indicating very good predictive accuracy [6].

The STS Score is widely used to assess risk in cardiac surgery and has high accuracy in predicting short- and long-term mortality risk [3,4]. The score also provides risk prediction for outcomes such as stroke, length of hospital stay, and renal failure, making it an important tool in cardiac surgery risk management. The STS Score is calculated through the STS website and has been continually improved to provide more accurate predictions over time [24]. However, limitations of surgical risk scores such as the STS Score and the Japan Score have been identified [25]. The scores themselves are based on data from patients who have undergone surgery, and there is a lack of data on high-risk patients who are ineligible for surgery. In addition, the risk assessment tends to be lower for older patients [25].

In recent years, blood biomarkers (Table 7) measured preoperatively have been shown to be useful in predicting postoperative risk [8-10]. The plasma endothelin-1 level was shown to be a promising indicator for predicting the risk of postoperative AKI and heart failure in patients undergoing cardiac surgery [8]. Also, elevated plasma endothelin-1 levels have been reported to be associated with increased pulmonary hypertension, heart failure, and mortality [14]. A complex model, including both N-terminal pro-brain natriuretic peptide (NT-proBNP) and GDF-15, may play a supplemental role to support the treatment based only on BNP and better predict all-cause mortality and heart failure rehospitalization during a one-year follow-up [26]. Our previous study showed that preoperative serum levels of GDF-15 and endothelin-1, as well as intraoperative factors such as cardiopulmonary bypass time, may help identify short-term surgical risk for patients undergoing cardiovascular surgery [9]. Furthermore, this study found that serum GDF-15 levels were associated with the STS Score, and serum levels of GDF-15 and endothelin-1 were associated with the Japan Score.

**Table 7:** The biomarkers in this study GDF, growth differentiation factor; STS, the Society of Thoracic Surgeons

Biomarkers	With respect to STS Score and Japan Score	Normal reference values
GDF-15	Related to STS Score and Japan Score	0.34-1.06 ng/mL
Endothelin-1	Related to Japan Score	0.47-2.00 pg/mL
Adiponectin	Not related	0.87-21.42 μg/mL

In this study, higher serum adiponectin levels were associated with higher STS Scores and Japan Scores. However, the association between serum adiponectin levels and STS Scores and Japan Scores disappeared on multivariate analysis using serum levels of GDF-15 and endothelin-1 as covariates. Adiponectin has been reported to improve insulin resistance by activating adenosine monophosphate-activated protein kinase in skeletal muscle and liver, thereby promoting fatty acid burning and glucose uptake [27]. Also, adiponectin was shown to suppress atherosclerosis, as adiponectin-deficient mice show increased vascular inflammatory intimal thickening [28]. Furthermore, adiponectin was found to have anti-inflammatory and anti-apoptotic effects and to reduce cardiovascular oxidative stress [29]. Regardless of these apparent positive effects, in recent studies, it has been reported that in the pathogenesis of cardiovascular disease, high levels of adiponectin do not necessarily represent better clinical outcomes [21,30], as was the case in this study. Furthermore, elevated circulating adiponectin levels have been reported to be a paradoxically negative prognostic determinant in patients with advanced heart failure, which reflected underlying disease status, BNP levels, and systemic inflammation [21]. The present study showed a loss of the association between adiponectin and surgical risk scores in the multivariate analysis, which was consistent with the findings of that report. In this study, we showed that the Japan Score is more strongly associated with heart failure than the STS Score. One possible mechanism for this is that the Japan Score includes a heart failure item.

Strengths and limitations

 As far as we know, this study, for the first time, showed that endothelin-1 gave the edge to the Japan Score over the STS Score in heart failure, and BNP, determining endothelin-1, was deemed to have an adverse cardiac effect. As a limitation, firstly, this study was performed at a single institution with a rather small number of patients. Thus, future large-scale studies of surgical risk scores at multiple centers might be needed. Secondly, because of the retrospective nature of the present study, future prospective studies might also be needed.

## Conclusions

Traditional biomarkers such as BNP and NT-proBNP have been widely adopted for their cost-effectiveness and ease of access. However, the rise of novel biomarkers such as GDF-15 and adiponectin has shown promising results, offering superior sensitivity and specificity, although these novel biomarkers are not more cost-effective than traditional biomarkers. These new biomarkers enhance diagnostic accuracy, risk stratification, and prognostic evaluation in heart failure patients. Serial measurement of multiple biomarkers among acute heart failure patients is promising and provides a better prognostic value than the measurement of a single marker at a single time point. A complex model, including both NT-proBNP and GDF-15, may play a supplemental role to support the treatment based only on BNP and better predict all-cause mortality and heart failure rehospitalization. In this study, the Japan Score was suggested to be a better predictor of risk, especially related to heart failure, than the STS Score in patients undergoing cardiovascular surgery. The combination of these surgical risk scores and serum endothelin-1 may improve early detection of heart failure in cardiac surgery and enable the determination of treatment strategies according to the risk of each patient.
